# Using NextRAD sequencing to infer movement of herbivores among host plants

**DOI:** 10.1371/journal.pone.0177742

**Published:** 2017-05-15

**Authors:** Zhen Fu, Brendan Epstein, Joanna L. Kelley, Qi Zheng, Alan O. Bergland, Carmen I. Castillo Carrillo, Andrew S. Jensen, Jennifer Dahan, Alexander V. Karasev, William E. Snyder

**Affiliations:** 1 Department of Entomology, Washington State University, Pullman, Washington, United States of America; 2 School of Biological Sciences, Washington State University, Pullman, Washington, United States of America; 3 Department of Bioinformatics and Biostatistics, University of Louisville, Louisville, Kentucky, United States of America; 4 Department of Biology, Stanford University, Stanford, California, United States of America; 5 Northwest Potato Research Consortium, Lakeview, Oregon, United States of America; 6 Department of Plant, Soil and Entomological Sciences, University of Idaho, Moscow, Idaho, United States of America; Natural Resources Canada, CANADA

## Abstract

Herbivores often move among spatially interspersed host plants, tracking high-quality resources through space and time. This dispersal is of particular interest for vectors of plant pathogens. Existing molecular tools to track such movement have yielded important insights, but often provide insufficient genetic resolution to infer spread at finer spatiotemporal scales. Here, we explore the use of Nextera-tagmented reductively-amplified DNA (NextRAD) sequencing to infer movement of a highly-mobile winged insect, the potato psyllid (*Bactericera cockerelli*), among host plants. The psyllid vectors the pathogen that causes zebra chip disease in potato (*Solanum tuberosum*), but understanding and managing the spread of this pathogen is limited by uncertainty about the insect’s host plant(s) outside of the growing season. We identified 1,978 polymorphic loci among psyllids separated spatiotemporally on potato or in patches of bittersweet nightshade (*S*. *dulcumara*), a weedy plant proposed to be the source of potato-colonizing psyllids. A subset of the psyllids on potato exhibited genetic similarity to insects on nightshade, consistent with regular movement between these two host plants. However, a second subset of potato-collected psyllids was genetically distinct from those collected on bittersweet nightshade; this suggests that a currently unrecognized source, i.e., other nightshade patches or a third host-plant species, could be contributing to psyllid populations in potato. Oftentimes, dispersal of vectors of pathogens must be tracked at a fine scale in order to understand, predict, and manage disease spread. We demonstrate that emerging sequencing technologies that detect genome-wide SNPs of a vector can be used to infer such localized movement.

## Introduction

Herbivores often move among host plant species, driven by their need to evade and detoxify plant defenses, balance nutritional requirements that cannot be met by single plants, and/or track spatiotemporal variation in plants’ resource quality [1, 2]. At the broadest scale, herbivores may traverse thousands of kilometers, tracking host-plant availability across seasons (e.g. [3–6]) or due to varying rainfall and wind patterns (e.g. [7, 8]). At a finer scale, herbivores often move among host-plant species within a habitat while tracking host-plant phenology, as different host-plant species go through seasonal changes in nutritional value and/or ability to physically or chemically defend themselves [9–11]. When herbivores act as vectors of plant pathogens, these movements can have particularly dramatic effects on host plants; herbivores can initiate pathogen outbreaks even when herbivore densities are too low to inflict appreciable direct damage [12–14]. Oftentimes, a detailed understanding of movement of vectors among host plant species, or within stands of the same species, is critical for predicting patterns of disease spread (e.g. [14–16]).

When herbivores are relatively large, or the distances covered are relatively small, physically marking and tracking individual herbivores can be an effective way to unravel patterns of host-plant switching [17–19]. However, when this is impossible or impractical, patterns of interrelatedness among herbivores can be used to infer likely movement patterns. Molecular techniques, including protein and microsatellite DNA markers, were among the first genetic tools used to infer gene flow and thus herbivore dispersal [20–22]. However, developing a sufficiently large set of markers to delineate localized movement can be time consuming and expensive, or even impossible when there are few microsatellites in the genome [23–25]. Recently, restriction-site associated DNA (RAD) markers have been used to overcome these limitations by allowing quick detection of single nucleotide polymorphisms (SNPs) across focal organisms’ entire genomes. RAD-based approaches have proven powerful in tracking genetic differentiation across landscapes (e.g. [26, 27]), but the relatively high DNA-volume inputs required has thus far limited their use to larger-bodied organisms. Because of their small body sizes, many herbivorous insects that feed heavily on plants (and/or vector key plant pathogens) have thus far been outside the reach of these approaches.

Here, we explore the use of Nextera-tagmented reductively-amplified DNA (“NextRAD”) sequencing to infer movement among host plant species by a winged, small-bodied insect, the potato psyllid (*Bactericera cockerelli*). The psyllid is the vector of the bacterium (*Candidatus* Liberibacter solanacearum) that causes zebra chip disease in cultivated potato (*Solanum tuberosum*) [28], whose spread has endangered potato production in several parts of the United States of America (e.g. [29]). In the northwestern U.S., it has been proposed that potato psyllids transmit the zebra chip pathogen as the insects migrate from the perennial solanaceous weed bittersweet nightshade, *Solanum dulcumara*, to annually-cultivated *S*. *tuberosum* fields each year [30]. However, movement of potato psyllids from bittersweet nightshade to potato has never been directly demonstrated, hindering any ability to understand, predict, or manage zebra chip outbreaks [30]. Indeed, a wide variety of plant species other than bittersweet nightshade have been proposed to be the true source of psyllids (and perhaps also the zebra chip pathogen) that colonize potato fields [31]. Existing molecular tools for dividing psyllids into geographically-separated genetic groups, based on sequence variation within the cytochrome *c* oxidase I (COI) gene, are too limited to reveal genetic subpopulations at a fine-enough scale to identify gene flow among host plants [30]. NextRAD sequencing overcomes these limitations by fragmenting and ligating adaptor sequences to genomic DNA via engineered transposomes. Critically, NextRAD requires less than 50 ng of DNA [32, 33], making it possible to generate sequence data from organisms far smaller than was possible with the original RAD sequencing approaches. Using this technique in combination with high-throughput sequencing generates a large number of markers, greatly facilitating examination of the genomic variation of psyllid populations and individuals. Thus, we could assess whether bittersweet nightshade could be the sole source of potato psyllids colonizing potato fields, or whether instead other non-crop host species might need to be identified.

## Materials and methods

**Fig 1:**
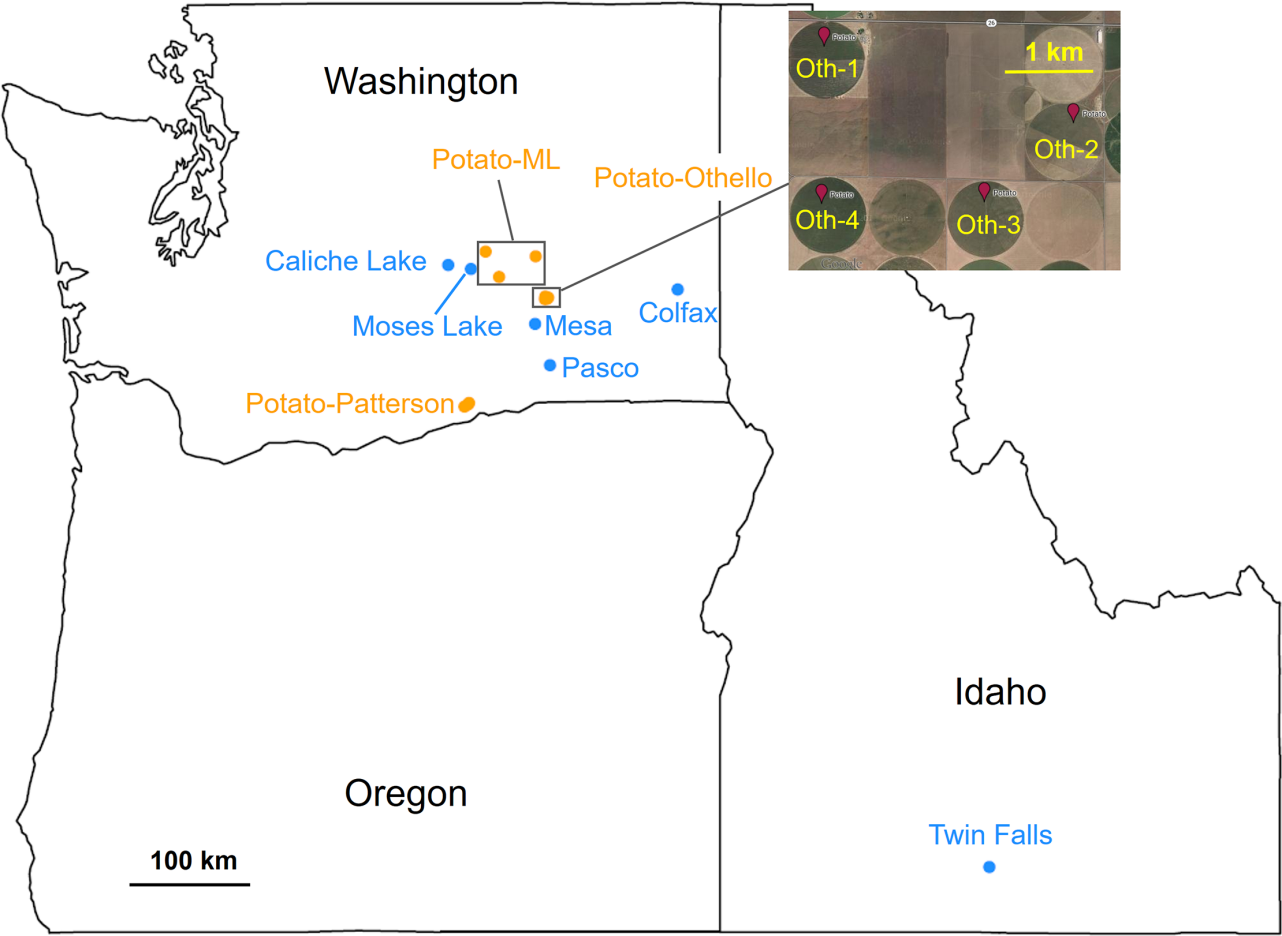
Potato psyllid collection sites across the states of Washington and Idaho, United States of America. Potato psyllids were collected from bittersweet nightshade patches (blue circles) or potato fields (yellow circles). See Table 1 and S1 Table for detailed information about each population.

Our project included regional sampling of spatially-dispersed herbivore populations on two host plant species, followed by sequencing the insects to infer population interrelatedness. First, over two years, we collected potato psyllids from bittersweet nightshade patches located throughout much of the potato-growing region of east-central Washington State (USA); in one of these years, we also collected psyllids from production potato fields across this same region (Fig 1). Additionally, we collected psyllids from a nightshade patch located in southern Idaho (Fig 1) to serve as a geographically-distinct outgroup. A subsample of the psyllids collected from nightshade patches (up to 10 psyllids per sampling date), and all psyllids collected from potato fields, were then sequenced using the NextRAD approach; this allowed us to identify variant sites throughout the psyllid genome. We then used multiple population-genetic approaches to determine population structure among psyllids collected from the two host plants, to infer whether they are composed of a single interbreeding population or instead include members of genetically-distinct sub-populations. Each of these project sub-components are detailed below.

### Potato psyllid sampling and sequencing

Our study did not involve any endangered or protected insect species, and no specific permits were required. For the psyllids collected from private land, we obtained the permission from the land owners. Potato psyllids were collected from six bittersweet nightshade patches and ten potato fields in the U. S. states of Washington and Idaho (Fig 1 and Table 1), over two growing seasons, using a suction sampling device (see [34, 35]). Sites were chosen to cover the majority of the potato-growing region in east-central Washington, with the Idaho site serving as a geographically-distant outgroup, and were sampled periodically over the 2012 and 2013 growing seasons (Fig 1 and S1 Table). Psyllids were placed on dry ice immediately following collection, and were stored in 95% ethanol upon arrival in the laboratory. For each sampling date, on each host plant and at each location, four to ten intact adult psyllids were randomly selected for DNA extraction and sequencing (Table 1 and S1 Table). In total, we processed 285 psyllids for NextRAD sequencing.

**Table 1:** Sampling locations and sampling dates of potato psyllids *Bactericera cockerelli* in the states of Washington and Idaho, United States of America.

Sampling ID	Host plants	Coordinates	# of psyllids sequenced	# of sampling dates
Caliche Lake	Nightshade	47° 1'54''N, 119°55'40"W	18	2
Colfax*	Nightshade	46°50'51"N, 117°28'44" W	31	4
Mesa*	Nightshade	46°35'18"N, 119° 0'1"W	66	7
Moses Lake*	Nightshade	47° 0'7"N, 119°41'5"W	60	6
Twin Falls	Nightshade	42°29'57"N, 114° 9'15"W	50	5
Pasco	Nightshade	46°16'39"N, 118°50'29"W	10	1
ML-1	Potato	46° 56' 31"N, 119° 23' 4" W	5	2
ML-2	Potato	47° 7' 58"N, 119° 31' 43"W	1	1
ML-3	Potato	47° 5' 50"N, 118° 59' 41" W	1	1
Othello-1	Potato	46° 47' 40"N, 118° 53' 30"W	11	2
Othello-2	Potato	46° 47' 12"N, 118° 51' 41"W	10	2
Othello-3	Potato	46° 46' 40"N, 118° 52' 20"W	6	1
Othello-4	Potato	46° 46' 29"N, 118° 53' 44"W	10	1
Patterson-1	Potato	45° 57' 57"N, 119° 45' 15"W	4	1
Patterson-2	Potato	45° 58' 55"N, 119° 42' 58"W	1	1
Patterson-3	Potato	45° 59' 36"N, 119° 42' 19"W	1	1

* indicated that location was sampled for both 2012 and 2013, the other locations were sampled either in 2012 or 2013.

To begin DNA extraction, individual psyllid adults were placed into separate microcentrifuge tubes with 150 μl tissue lysis buffer (10mM Tris pH = 8; 50mM Ethylenediaminetetraacetic acid; 200mM NaCl; 1% (w/v) Sodium dodecyl sulfate; we found this homemade lysis buffer to be more efficient in breaking down the insect exoskeleton than the buffer in the commercial kit), and ground for 1 minute using a pestle driven by a handheld electric mixer. Thereafter, DNA extraction was conducted following the instructions of the Qiagen DNeasy Blood & Tissue Kit (Qiagen, Valencia, CA). DNA was eluted in 100 μl 10 mM Tris-HCl buffer (pH = 8). The quantity of DNA extracted from each insect was measured using a Qubit 2.0 Fluorometer (Life Technologies, Grand Island, NY).

DNA samples were sent to SNPsaurus LLC (Eugene, OR) to generate NextRAD libraries and perform sequencing. To construct DNA libraries, genomic DNA (~10 ng) was first fragmented with the Nextera reagent (Illumina, San Diego, CA), which also ligated short adapter sequences to the ends of the fragments (S1 Fig). DNA fragments were then amplified with two primers matching adaptor sequences, with one of the primers extending an additional nine nucleotides (GTGTAGAGC) as the selective sequence at the 3’ end (S1 Fig). Thus, only fragments that could be hybridized to the selective sequence were efficiently amplified. The libraries were sequenced on an Illumina HiSeq2000 with 1x100 bp configuration to generate ~65X coverage.

For each insect, an aliquot of the same DNA sent for NextRAD sequencing was used for COI “haplotyping” through high-resolution melting analysis [36]. This is an approach used to delineate genetic subgroups of potato psyllids at a coarser, continental scale (i.e., the “Central”, “Western”, and “Northwestern” haplotypes typical of different sections of North America; [36]) relative to the fine-scale genetic differentiation within our study region that NextRAD provided (see below).

### Sequence alignment, variant calling, and filtering

Quality trimming of raw reads and variant calling were performed by SNPsaurus. Trimmomatic [37] was used to remove the Nextera adapters and low quality reads (Phred quality score < 20). Thereafter, reads of all psyllids were pooled and aligned to each other to form allelic clusters (> 95% identity) using custom scripts, and the read with the highest count in the population was chosen as a reference contig. In order to identify these contigs based on gene homology, all 23,191 contigs were queried to the NCBI reference sequence (RefSeq) database [38] using BLASTN [39] with an e-value cutoff of 0.0001. Subsequently, reads from each sample were aligned to the reference using the BWA-mem algorithm (parameters: -B 3 -O 4 -k 13) of the Burrows-Wheeler Aligner [40]. Variant sites were called by SNPSaurus using the mpileup and bcftools algorithms in SAMtools [41].Bcftools uses a statistical approach to call variants. With the conservative parameters used in this study, it was more likely to call a heterozygote with low read numbers as a homozygote than call a sequencing error an allele. Thereafter, we employed PLINK (v1.90; [42]) to calculate heterozygosity, allele frequencies and the missing data rate. Loci that were missing in > 5% of individuals (2,400 loci) and individuals with > 10% missing loci (four individuals) were excluded from the dataset. In addition, we removed loci with observed heterozygosity > 0.5, excluded loci with minor allele frequency (MAF) < 0.05, and removed all the indels. We randomly sampled one variant from each contig to assure the loci were mostly independent. We used PLINK [42] to report the p-value of Hardy-Weinberg Equilibrium (HWE) tests for each locus within sampled populations (with samples grouped by site and date of collection). HWE tests were applied only to the loci with no missing genotypes, and we only analyzed populations with sample size ≥ 8. We tested the outlier loci using three programs: OutFLANK [43], LOSITAN [44] and BayeScan [45]. For LOSITAN and BayeScan, the default setup was used, and for OutFLANK, q-value was set to 0.05.

### Neighbor-joining tree construction

First, we used the neighbor-joining clustering technique in order to visually describe interspersion and/or separation of psyllids based on the host plant, site, and date from which the insects were collected. We calculated genetic similarity (proportion of shared alleles) between all pairs of individuals using the “—distance square 1-ibs” option in PLINK (v1.90; [42]). We then constructed an unrooted neighbor-joining tree [46] from the pairwise genetic distances using the “nj” function in the R package ape (v3.3;[47]) and obtained support values by randomly resampling variants with replacement 500 times (bootstrapping) in R and the R package ape [47].

### Clustering analysis and population structure

We next used ADMIXTURE [48] to delineate genetically-distinct groups within our psyllid collections, searching for the number of genetic lineages that best described the data. We increased the pre-defined number of ancestral populations (*K*) from *K* = 1 to *K* = 20. Ancestry coefficient matrices from 50 replicated runs were aligned and averaged using the program CLUMPAK [49]. Because gene flow among sites could occur throughout our study region, and because the ancestry and lineage of all psyllid samples was unknown, we conducted ADMIXTURE analysis in the “unsupervised” mode without providing any sampling information, and we identified the best *K* value as the run with the lowest cross-validation error [48].

As a complementary approach examining the genetic population structure of psyllids separated by hosts, time and space, we also conducted principal component analysis (PCA) using the smartpca algorithm from EIGENSOFT (v6.0.1; [50]).

### *F*-statistics and spatiotemporal separation

Third, as a means of inferring how patterns of psyllid interrelatedness differ through time and space, we calculated fixation indices (*F*_ST_) among pairs of collection sites and dates. We first estimated the inbreeding coefficient (*F*_IS_), based on Nei [51], of psyllid collections that included ≥ 4 psyllids (S1 Table). Next we estimated population differentiation (*F*_ST_) between pairs of collections that included ≥ 4 psyllids (S1 Table), based on the equations described in Weir and Cockerham [52]. First, we employed linear models to examine the relationship between geographic distance separating psyllid populations and their degree of genetic interrelatedness, working with insects collected between August, 2012 and October, 2013 (this was the time window during which the most sites were sampled roughly synchronously). Second, within sites sampled repeatedly through time, we examined the relationship between degree of temporal separation and the degree of genetic divergence.

### Analysis of Molecular Variance (AMOVA)

Fourth, to determine the relative importance of host, time, ADMIXTURE clusters, and spatial separation among the Washington samples, we ran two AMOVAs [53] using the poppr [54, 55] and ade4 [56] R packages. Collection population (the combination of location and time) was nested within either the ADMIXTURE population assignment or the host plant species. We used the *K* = 3 run to determine the ADMIXTURE assignment and assigned samples based on the population with the greatest ancestry fraction. We tested significance for the host AMOVA using 1000 random permutations; significance was not assessed for the ADMIXTURE cluster run because testing the significance of clusters defined by exploratory analyses on the same dataset is circular, and produces meaningless *p*-values [57].

## Results

### Sequencing

On average 400 Megabases of sequence data, equivalent to ~ 2.7 million 100 bp reads, were obtained from each psyllid NextRAD library. As no reference genome was available, a *de novo* assembly was constructed using a custom script from our sequencing service provider, SNPsaurus. We searched for homologs of each contig in the assembly in the RefSeq database [38]. However, only 2.7% (643 of 23,191) of the contigs returned homologies and most of these contigs aligned with sequences of the Asian citrus psyllid, *Diaphorina citri*; *D*. *citri* is the most-closely-related species to the potato psyllid that has been sequenced [58]. We identified 8,443 variants by aligning cleaned reads of each sample back to the *de novo* assembly. After removing indels, loci with a high missing data rate, loci with high heterozygosity (> 0.5), and randomly sampling independent loci (described in Material and Methods), we included 1,978 loci in the downstream analyses (S2 Table). Within the 1,978 loci, no locus failed the HWE test (p-value < 0.001) and none of the loci were detected to be under selection by all three programs.

Our COI-haplotyping revealed that all 285 potato psyllids belonged to the “Northwestern” COI-haplotype that is typical of the region where our work was conducted (e.g., [36]). As described below, we used several methods, including neighbor-joining clustering, ADMIXTURE, PCA, and AMOVA, to characterize finer-scale population structure using the NextRAD variants.

### Neighbor-joining tree

**Fig 2:**
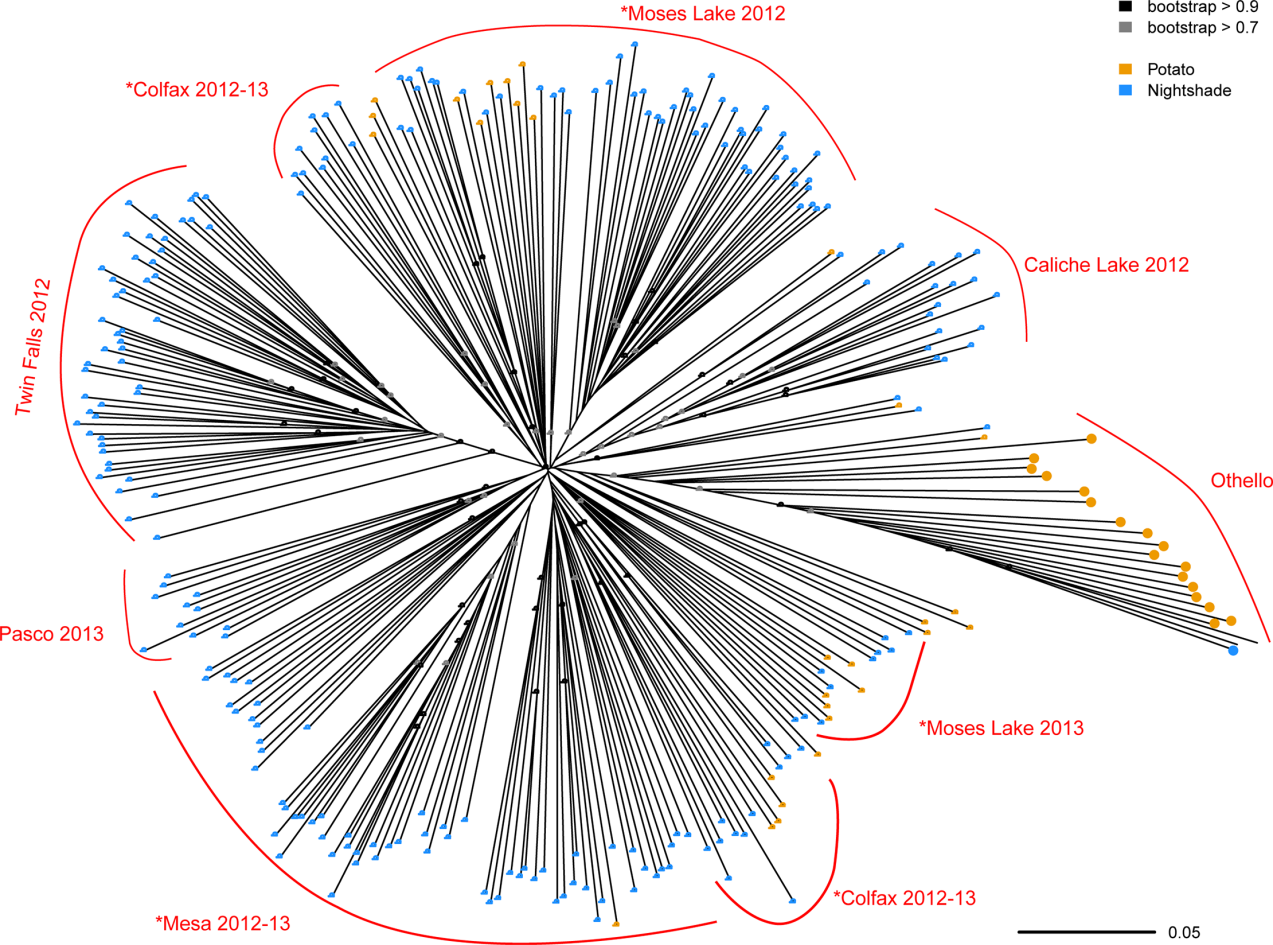
Unrooted neighbor-joining tree for potato psyllids collected from bittersweet nightshade patches (blue circles) or potato fields (yellow circles). The neighbor-joining tree was constructed using proportion of shared alleles. Psyllids from Potato-ML and Potato-Patterson were not labeled as they intermixed with psyllids from nightshade sites. An asterisk (*) indicates three nightshade locations with samples spanning two years. Samples with enlarged circles were grouped into a genetically distinct group in ADMIXTURE (shown in purple bars in Fig 3).

Clustering using the neighbor-joining method indicated that samples taken from the geographically isolated bittersweet nightshade patch near Twin Falls, Idaho, formed a cluster separate from all other psyllids that we collected from either of the two host plants in Washington (Fig 2). Otherwise, psyllids collected from potato fields in Washington (ML and Patterson populations) were generally interspersed with insects collected from bittersweet nightshade patches in that same state, particularly the Colfax and Moses Lake sites (Fig 2). An exception to this broader pattern was a group of psyllids collected from a suite of potato fields near Othello, Washington (enlarged circles in Fig 2, Table 1 and S1 Table); this group of potato-collected psyllids fell out in a distinct cluster separate from any psyllids collected from any other potato field (Fig 2). Note that the terminal branch lengths were quite long for this potato-collected “Othello” cluster, indicating substantial genetic variation among individual psyllids in the cluster (Fig 2). Potato psyllids collected across two years at the Moses Lake bittersweet nightshade site clustered separately, suggesting genetic differentiation between years. In contrast, the insects from the Mesa site with multi-year collections clustered together (Fig 2). Psyllids from Colfax of two years were placed into different clades, but the separation was not temporal related, suggesting genetic divergence among psyllid individuals within a year.

### Admixture and PCA

**Fig 3:**
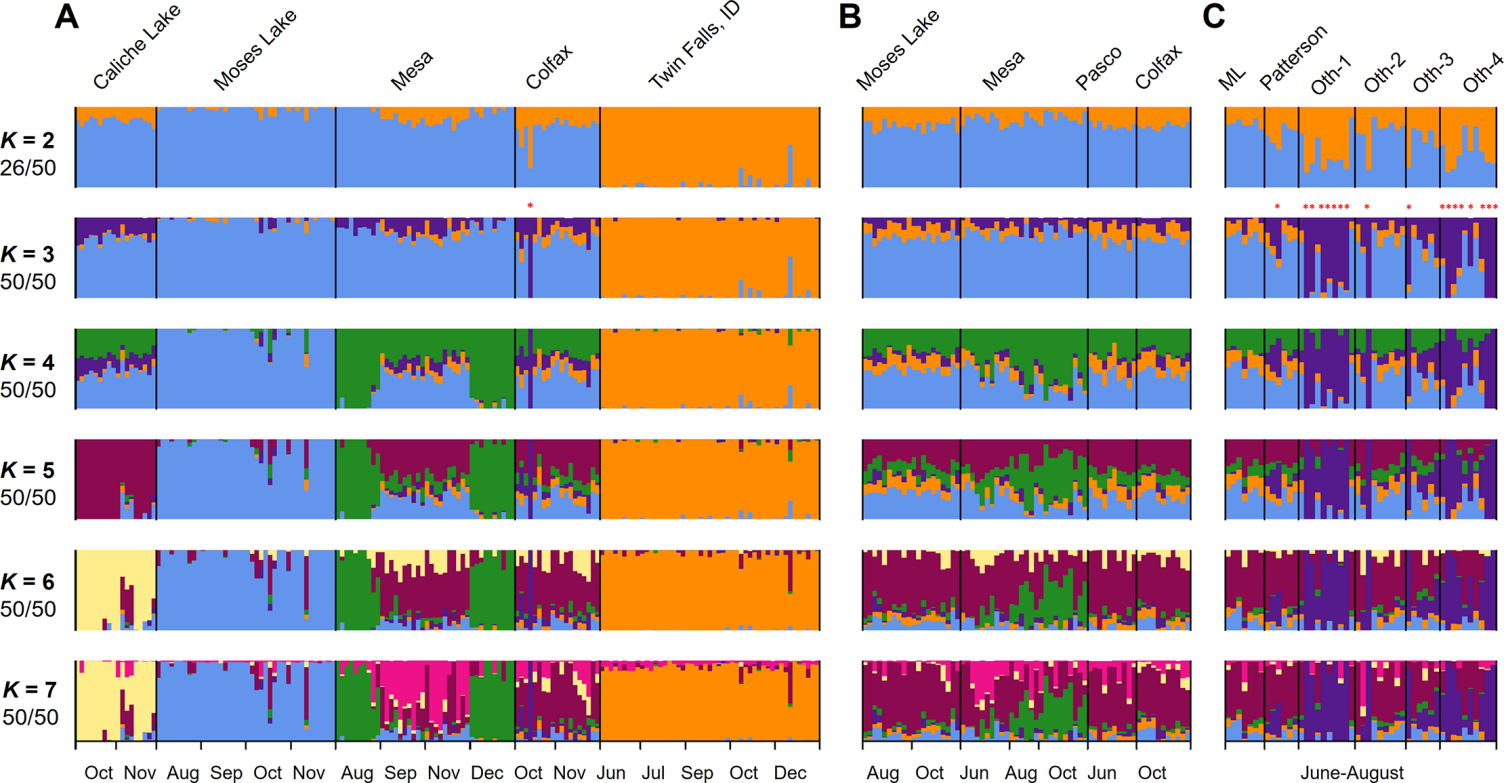
Estimated ancestry of potato psyllids collected from bittersweet nightshade patches and potatoes. Potato psyllids were collected from bittersweet nightshade patches in (A) 2012, (B) 2013, and from potato fields in (C) 2013. The number of ancestral populations (*K*) ranged from *K* = 2 to *K* = 7, and only the grouping pattern that represents the majority (> 50%) of the runs were presented. Numbers below each *K* indicate the number of runs (of 50 runs) showed the representative grouping. Each vertical bar represents a psyllid individual. * indicate “purple cluster” (assigned by majority-rule at *K* = 3), a genetically distinct group which was further analyzed in AMOVA.

In general, the population structure identified by ADMIXTURE (Fig 3) was consistent with the results of the neighbor-joining method: Idaho and Washington samples were differentiated, there was a group of psyllids, mostly from potatoes at the Othello site, that were also distinct, and there was otherwise little evidence for distinct potato and nightshade populations. In particular, At *K* = 2 (i.e. ADMIXTURE was constrained to split the samples into two groups), we saw separation of the psyllids of the most geographically-distinct population, the single nightshade patch in southern Idaho, from the majority of the psyllids collected in Washington. However, we noticed that in half of the 50 runs, some psyllids collected from Othello were grouped with the populations in Idaho (Fig 3). Interestingly, in 13 out of 50 runs, these Othello psyllids showed the opposite pattern, and they shared more similarity with the other psyllid populations in Washington (S3 Fig). The next group to separate from the others, at *K* = 3, did not reflect host plant species; rather, these were the same group of potato-collected psyllids, from potato fields near Othello, WA, identified by the neighbor-joining method as being genetically distinct (Figs 2 and 3). This grouping persisted through higher *K* values. At *K* = 4 some of the psyllids collected from bittersweet nightshade at Mesa and Caliche Lake were placed in a separate group; there was also potential genetic turnover for potato psyllids collected at the Moses Lake and Mesa sites between the two years during which those bittersweet nightshade patches were sampled, whereas the Colfax site exhibited constant genetic makeup across the two years (Fig 3). *K* values of 5 through 7 identified relatively modest genetic divisions within sites and host-plant species. We found that running ADMIXTURE with *K* = 9 minimized the cross-validation error (S2 Fig). However, there were three and five grouping patterns among the 50 runs at *K* = 8 and *K* = 9, respectively, none of the patterns represented the majority of the runs, and many individuals were highly admixed (S3 Fig). Thus, the biological interpretation of the higher *K* values was not obvious and may not be very informative for understanding contemporary psyllid population structure.

**Fig 4:**
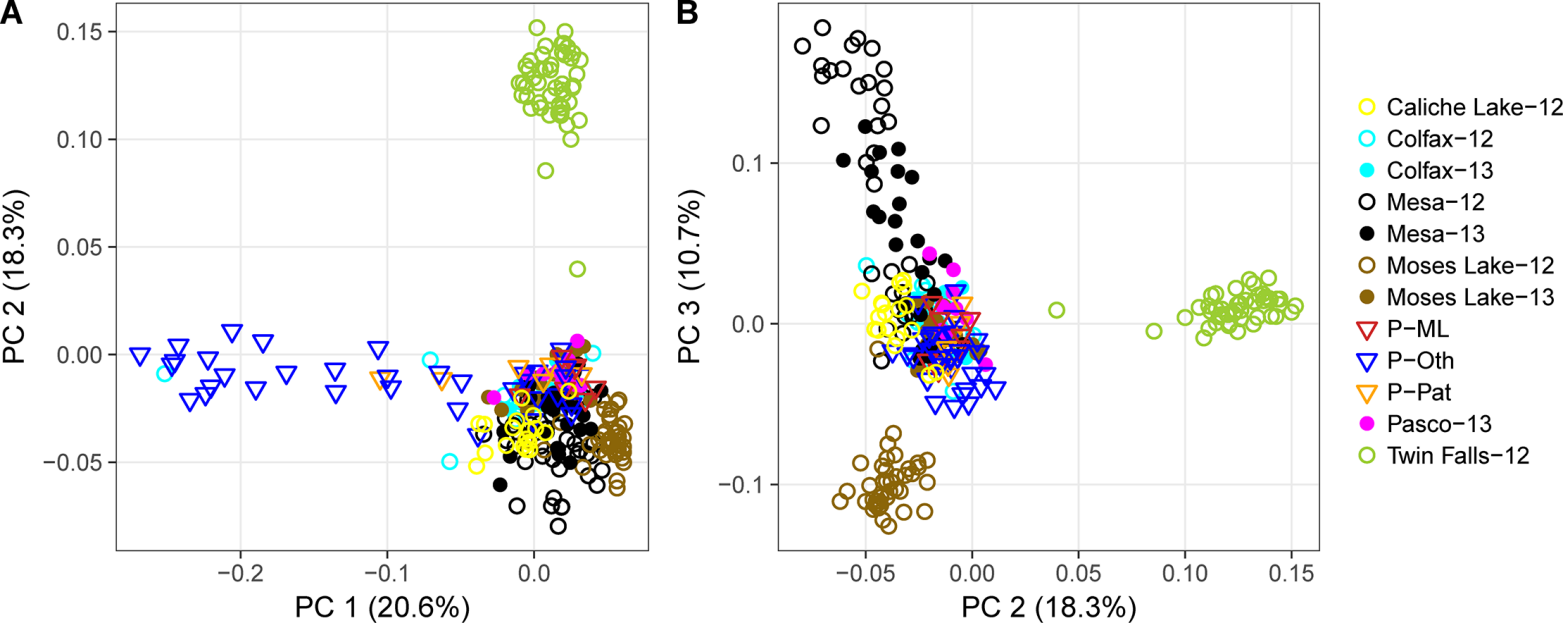
Principal component analysis (PCA) of genotypes of potato psyllids from nightshades and potatoes. (A). Principal component (PC) 1 and PC2. (B). PC2 and PC3. Psyllids were grouped by sampling sites (same color indicate same site), sampling year (non-filled symbol: 2012; filled symbol: 2013) and host plants (circle: nightshades, triangle: potatoes). Percentage accounted for overall genotype variability of each PC was indicated in the axis labels.

PCA revealed patterns similar to those detected with the other methods. Principal component (PC) 1 separated psyllids from Othello (dark blue triangles, Fig 4A) from those collected in other locations; this further supports the genetic distinctiveness of these psyllids (Fig 4A). Psyllids from Washington clustered together regardless their host plant species, and separated from Twin Falls populations collected in Idaho along PC2 (Fig 4B). Patterns of temporal variation for psyllids from the three nightshade patches with multi-year samples were more or less consistent with ADMIXTURE (Figs 3 and 4); specifically, samples from Mesa collected in different years largely overlapped, and samples from Colfax did not show temporal related separation. Conversely, the 2012 and 2013 samples from Moses Lake exhibited clear separation. Because the separation between the “third” admixture group and the other WA samples seemed much stronger than the separation between potato and nightshade psyllids, or among other sampling sites, we performed additional analyses on the samples assigned to that “third” cluster.

### *F*-statistics and geographic separation

**Fig 5:**
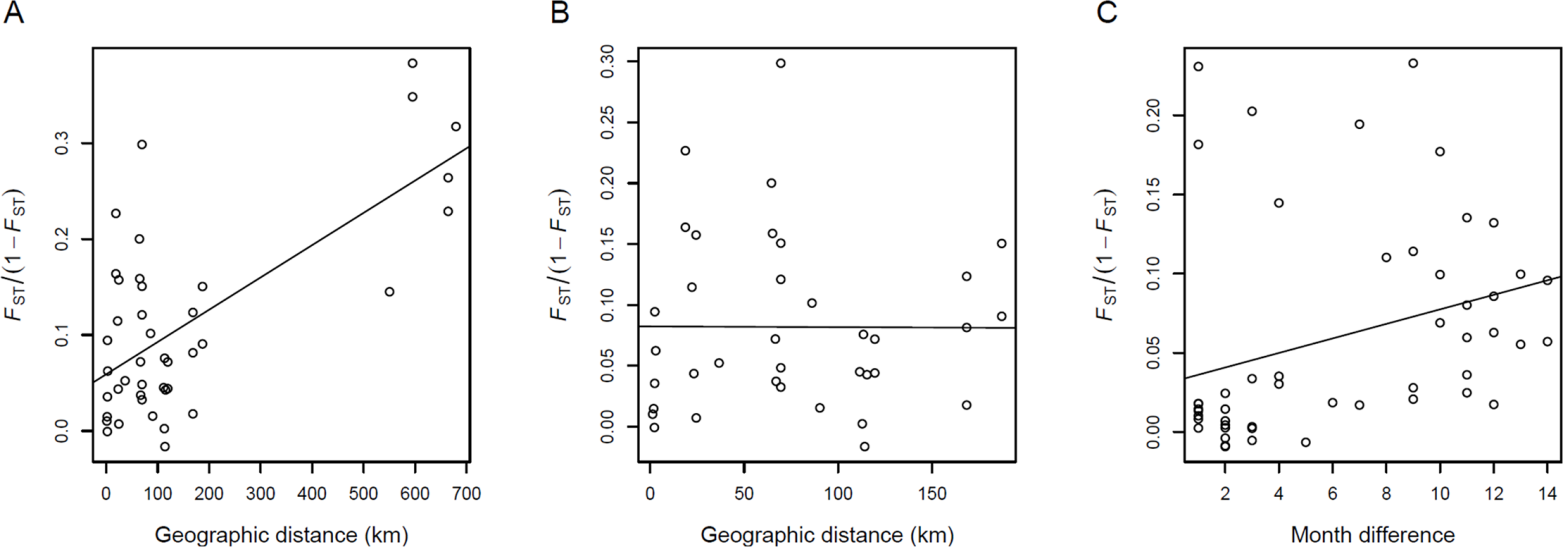
*F*_ST_ of potato psyllid populations separated spatiotemporally. (A) Regression of *F*_ST_ versus degree of geographic separation between pairs of psyllid populations differing in distance (but collected during the same month) for all population pairs across sampling dates from August, 2012 through October, 2013, and (B) for the same pairs of populations without the Twin Fall, ID, outgroup. (C) Regression of *F*_ST_ values for pairwise populations only differing in time (but collected at the same locations) across months.

The inbreeding coefficients (*F*_IS_) of psyllids collected from potatoes (mean *F*_IS_ = 0.196) were markedly higher than the *F*_IS_ of psyllids from bittersweet nightshade patches (mean *F*_IS_ = 0.08, Wilcoxon signed-rank test, *p*-value = 0.0004, S4 Fig), suggesting smaller effective psyllid populations in potato fields. We then investigated the correlation of population differentiation (*F*_ST_) and geographic distance. As described above, we included in these analyses the potato psyllids collected from August 2012 through October 2013, when insects were collected roughly synchronously across all sampled sites. We found a statistically significant relationship between increasing degree of geographic separation and increasingly-large *F*_ST_ when the single Idaho bittersweet nightshade patch, the most-distant site, was included in the analysis (R^2^ = 0.46, df = 40, *p-*value = 8.8e-07; Fig 5A). However, when that single-most-distant site was dropped from the analysis, this significant relationship disappeared (R^2^ < 0.1, df = 34, *p-*value = 0.978; Fig 5B).

We found a weak, but statistically-significant, correlation between within-site *F*_ST_ values and the duration of time between collections (R^2^ = 0.09, df = 48, *p-*value = 0.031; Fig 5C); this suggests increasing genetic differentiation within sites across time. In contrast to the relatively weak overall trend, *F*_ST_ of populations separated temporally at the Mesa and Moses Lake nightshade sites exhibited relatively large genetic changes through time. At the Mesa site within 2012, the *F*_ST_ between August and September populations was 0.154, compared to an *F*_ST_ between September and November of only 0.002 (S3 Table). At the Moses Lake site, the *F*_ST_ of any pairwise comparison between sampling dates within the same year was ≤ 0.022. In contrast, the *F*_ST_ of pairwise populations spanning two years was much higher (S4 Table), in agreement with the results of the neighbor-joining tree and ADMIXTURE analyses. Psyllids at the Colfax nightshade site were more residential, as the *F*_ST_ of temporally-separated populations was consistently low (between 0.013 and 0.024 across the two years).

Psyllids from Othello were evidently distinct from psyllids collected elsewhere (more psyllids were assigned to purple group in Fig 3 *K* = 3). Four populations from this region demonstrated different patterns when compared to psyllids from nightshade populations (S5 Fig). Specifically, *F*_ST_ of psyllids from Oth-1 versus psyllid populations across all the nightshade sites were generally greater than *F*_ST_ of Oth-2 versus all nightshade populations (the color was warmer of the Oth-1 column, S5 Fig), though psyllids of Oth-1 and Oth-2 were collected at the same month and two potato fields were only a few kilometers apart. Intriguingly, while comparing four psyllid populations from the Othello region to each other, the divergence was minor except Oth-1 versus Oth-2 (S5 Table).

### AMOVA

Because our sampling was hierarchical in space, and because we have several potential sources of genetic structure (space, time, and host plant), we performed AMOVAs to quantify how genetic variation is partitioned by each of these factors. Among the many Washington sites, regardless of whether the top level was host or ADMIXTURE cluster, the largest component of genetic variability was explained at the individual level (Table 2), with little genetic differentiation among sites within genetic clusters and among individuals within sites. Consistent with the clustering analyses, relatively little genetic variation (~4% of the total) was explained by host plant species, although the variation was significantly greater than zero (note that potato and nightshade populations were not always geographically adjacent). However, the clustering analyses (neighbor-joining, ADMIXTURE, and PCA) indicated that the strongest signal of population structure in WA comes from differentiation between some of the Othello (and one Colfax) samples—the “purple” group that appears at *K* = 3 in the ADMIXTURE analysis—and the other samples. When we made the top level in AMOVA the ADMIXTURE cluster (assigned by majority-rule at *K* = 3), we found that a moderate amount of the genetic variation (~18%) was partitioned by the separation between the “purple” or “third” cluster and the other WA cluster (see Materials and Methods). These results indicate that while overall there is little genetic differentiation between psyllids based on the host plant from which they were collected, the “third” genetic cluster identified by ADMIXTURE (Fig 3) is somewhat differentiated from the other Washington cluster.

**Table 2:** Results of two Analyses of Molecular Variance (AMOVAs) for samples within Washington. Populations were separated by sampling location and time.

% Variance	Φ-statistics	Component
With host as top level:		
3.8	Φ_host-total_ = 0.04^*^	Between hosts
9.0	Φ_pops-host_ = 0.09^*^	Within hosts, among populations
7.9	Φ_inds-pop_ = 0.09^*^	Within populations, among individuals
79.3	Φ_inds-total_ = 0.21	Within individual
With ancestry as top level:		
18.3	Φ_ancestry-total_ = 0.18	Between ADMIXTURE ancestry clusters
7.4	Φ_pops-ancestry_ = 0.09	Within ancestry clusters, among populations
5.4	Φ_inds-pop_ = 0.07	Within populations, among individuals
68.8	Φ_inds-total_ = 0.31	Within individual

Individuals were assigned to the ADMIXTURE cluster (*K* = 3) with the greatest proportion of ancestry.

* indicates a significantly greater variance than the expectation of randomly distributed variation at *p* < 0.001; Significance was not assessed for partitioning by ADMIXTURE cluster because the clusters were identified using the same data the AMOVA was performed on, the *p*-values would not be meaningful (e.g. [57]).

## Discussion

We took advantage of emerging NextRAD technology to examine interrelatedness of potato psyllids, vectors of a bacterium that causes zebra chip disease [59], collected from two host plant species. The insect has been suggested to overwinter on the perennial weed bittersweet nightshade before colonizing potato crops each summer, although this migratory linkage has never been demonstrated and many other putative non-crop hosts have been proposed [30, 31]. Multiple analyses indicated that the psyllids from bittersweet nightshade and potato crops formed regularly interbreeding populations not clearly separated by host plant (Table 2, Figs 2–4). For example, within our neighbor-joining tree (Fig 2) potato-collected psyllids were generally interspersed among psyllids collected from bittersweet nightshade patches in the same region. Likewise, our ADMIXTURE analysis showed potato- and nightshade-collected psyllids assigned to the same ancestral populations (Fig 3), and our PCA did not show substantial separation by host-plant species (Fig 4). All of these analyses are consistent with the small amount of genetic variance partitioned among host species in an AMOVA (Table 2). Overall, these results suggest that the psyllids found on potatoes during the growing season are very likely persisting on nightshade during the winter. Bittersweet nightshade is common in the Pacific Northwestern US, it grows near bodies of water and along fence lines in large stands [30]. From an applied perspective, this suggests that removal of weedy, invasive bittersweet nightshade plants from the landscape might reduce a key source of potato psyllids eventually colonizing, and perhaps bringing the zebra chip pathogen to, potatoes.

At the same time, there was a second, genetically distinct group of potato psyllids found in four potato fields near Othello, Washington (Fig 1, Table 1 and S1 Table), that strongly differed from these overall patterns. Insects from those fields fell out as a unique clade in our neighbor-joining tree (Fig 2), while clustering analysis suggested that some, though not all, of the Othello samples were genetically distinct from the other psyllids on both potato and nightshade in nearby fields and populations (purple bars in Fig 3). We assessed the magnitude of the genetic differentiation using AMOVAs and *F*_ST_: A comparison of AMOVAs run with either host or genetic cluster as the top-level population indicated that about five times more of the genetic variation could be explained by the separation between this unusual group and the other WA psyllids than could be explained by separation between host plant species and about twice as much variation as between sampling sites (Table 2). Furthermore, *F*_ST_ between the “purple cluster” in ADMIXTURE (*K* = 3–7, Figs 2 and 3) and the other Washington psyllids (0.18–0.20) was greater than that between the other Washington psyllids and those from the distant Idaho site (0.12–0.13, S6 Table), clearly indicating that this group is quite different from the other psyllids. There are at least two possible explanations for these findings. One is that there is a genetically-isolated sub-population of potato psyllids on bittersweet nightshade plants outside of our sampling network or that the distinct psyllids were moved in from a distant location. An intriguing, second possibility is that a third host plant species is the source of the unique potato-collected insects, with insects on that as-yet-unidentified plant species genetically isolated from those on bittersweet nightshade. It is unlikely that the differentiation we see is simply the result of a barrier to gene flow between Othello and the other sites because Othello is quite close to other sampling sites (Fig 1) and some of the Othello samples (Oth-2 and Oth-3 samples) cluster with the other WA psyllids (Figs 3 and 4, S5 Fig). We note that empirically testing these hypotheses will require sampling additional sites and plant species from Othello and the surrounding region. Many other putative potato psyllid host plant species have been suggested (e.g., other solanaceous weeds species, field bindweed *Convolvulus arvensis*, and matrimony vine *Lycium barbarum*; [31]). From an applied perspective, in turn, pest managers might consider the possibility that suppressing the exotic weed bittersweet nightshade might not entirely suppress regional potato-psyllid populations.

Several lines of evidence suggest that, despite the apparent stability of perennial bittersweet nightshade patches that may persist for decades, psyllids regularly move across the landscape. For example, we observed genetic turnover between (and even within) years at our Moses Lake and Mesa sites, as evidenced by genetic differentiation seen in the ADMIXTURE analysis from *K* = 4 through *K* = 7 (Fig 3). As a more general pattern, for pairings of collections within single sites but separated in time, we found that *F*_ST_ increased with increasing time between collection dates (Fig 5C). This suggests a general, although relatively modest, turnover in genetic makeup across sites through time that would be consistent with gene flow among sites (although micro-evolutionary adaptation to particular sites could also explain this result; e.g., [60]). Furthermore, we noted no relationship between degree of genetic divergence and geographic distance between sites within Washington (Fig 5B), consistent with a lack of strong barriers to gene flow among these sites. Geographic separation often strongly predicts genetic differentiation (e.g., [61, 62]), as indeed was the case when the most-distant Idaho site was included in analyses (Fig 5A). Perhaps these potato psyllids move readily among sites in the absence of significant physical (e.g., the Blue and Bitterroot mountain ranges) and biological (e.g., the relative dearth of irrigated agriculture) barriers separating the Washington and Idaho sites. It remains unclear if the insects are moving for nutritional reasons (e.g., [10,17,63]), perhaps related to the seasonal drought typical of the region that could render irrigated potato crops more attractive than water-stressed bittersweet nightshade plants. Of course, a wide variety of other biotic (e.g., [64]) and abiotic (e.g., [65]) factors are known to trigger dispersal of pathogen-vectoring herbivores in other systems.

While the findings we present here are specific to one particular plant-pathogen vector and a pair of its host plants, our approach could be widely applicable in other systems. Our study community is typical of many insect-vectored plant diseases, where a detailed understanding of population structure of vectors is critical for understanding, predicting and managing plant disease dynamics. For example, outbreaks of bean leaf roll viruses damaging to leguminous crops often depend upon movement of pea aphid (*Acyrthosiphon pisum*) vectors from alfalfa (*Medicago sativa* L.), a perennial host of both aphid and virus, onto peas [66]. This general movement of aphid between these two host plants has been documented using microsatellite markers in the pea aphid (e.g., [67]). However, sequencing approaches that detail SNPs across the genome, such as the NextRAD, could reveal fine-scaled population structure and thus infer aphid and virus movement among particular fields within a growing region (e.g., Figs 2 and 3). In turn, this degree of resolution could provide field-specific predictions of disease risk that benefit individual land-managers weighing treatment options. It is notable that fine-scale population structure of vectors is important not just for predicting movement of plant pathogens, but also when highly-mobile vectors spread vertebrate pathogens (e.g., [68]). Indeed, SNPs generated through RAD sequencing already hold promise for understanding relatively small-scale movement patterns of the mosquito *Aedes aegypti*, the vector of Dengue fever and other arboviruses [69]. This demonstrates the broad utility of these approaches for understanding the ecology of vector-transmitted diseases across diverse pathosystems.

The field of population genetics is increasingly making use of “Genotyping by Sequencing” which provides detailed information on genomic variation among individuals and populations. This approach has wide applicability in ecology and evolution, improving our understanding of site-specific adaptive evolution within species [70,71] and evolutionary origins and dispersal patterns of migratory species [72,73], while helping to associate loci with particular phenotypic traits [74,75]. As a powerful and the most-commonly-used approach, RAD sequencing has limitations. Key among these is the reliance on restriction enzyme digestion in the workflow, which limits the approach to use with vertebrates, or relatively large arthropods, from which a sufficiently-large quantity of DNA can be extracted from individuals (e.g., stickleback fish, [70]; land snails, [74]; butterflies, [76]). NextRAD substitutes transposomes for restriction enzymes, necessitating less DNA per sample and thus allowing the approach to be used with small amounts of DNA [32, 33]. This is critical, because relatively small-bodied insects make up a majority of the most injurious herbivores of plants in many natural and agricultural settings (e.g., aphids, fruit flies, and whiteflies), while small arthropods serve as key vectors of some of the most damaging animal and plant pathogens (e.g., mosquitoes, ticks, fleas, aphids, and thrips). Using the NextRAD approach, we were able to describe relatively detailed patterns of population structures of insects in a region. In turn, these patterns suggested local movement patterns of the vectors. We suggest that our work provides a model that may be of value in the many other systems where small-bodied insects move among host plants, and/or vector plant or animal pathogens.

## Supporting information

S1 Fig Workflow of NextRAD sequencing. (PDF)

S2 Fig Cross-validation (CV) error and standard error of ADMIXTURE runs. (PDF)

S3 Fig Estimated ancestry of potato psyllids from bittersweet nightshade patches and potatoes (*K* = 2, 8 and 9). (PDF)

S4 Fig Inbreeding coefficient of psyllid populations separated by plant hosts. (PDF)

S5 Fig Pairwise *F*_ST_ of psyllids separated on potato (Othello only) and nightshades. (PDF)

S1 Table Details of potato psyllid sampling. (PDF)

S2 Table Number of SNPs in each filtering step. (PDF)

S3 Table Pairwise *F*_ST_ of potato psyllids from nightshades at the Mesa site. (PDF)

S4 Table Pairwise *F*_ST_ of potato psyllids from nightshades at the Moses Lake site. (PDF)

S5 Table Pairwise *F*_ST_ of potato psyllids from potatoes at Othello. (PDF)

S6 Table Median pairwise genetic distance and *F*_ST_ among groups of psyllids designated following ADMIXTURE *K* = 3. (PDF)
