# Supplementary material for: Using NextRAD sequencing to infer movement of herbivores among host plants
Source: PLoS One. 2017 May 15;12(5):e0177742. doi: 10.1371/journal.pone.0177742 (PMC5432177; doi:10.1371/journal.pone.0177742)

S1 Fig. Workflow of NextRAD (Nextera-tagmented reductively-amplified DNA) sequencing. A small amount of DNA (~10 ng) is mixed with two engineered transposomes of Nextera reagents which tag as well as add short adaptors to genomic DNA. Sequencing primers Read 1 and Read 2 (light green and light blue bars), indices (orange and brown bars) and a customized selective primer (red bar: GTGTAGAGC) are added by a limited cycle of PCR to generate sequencing-ready fragments, which are compatible for Illumina platforms.

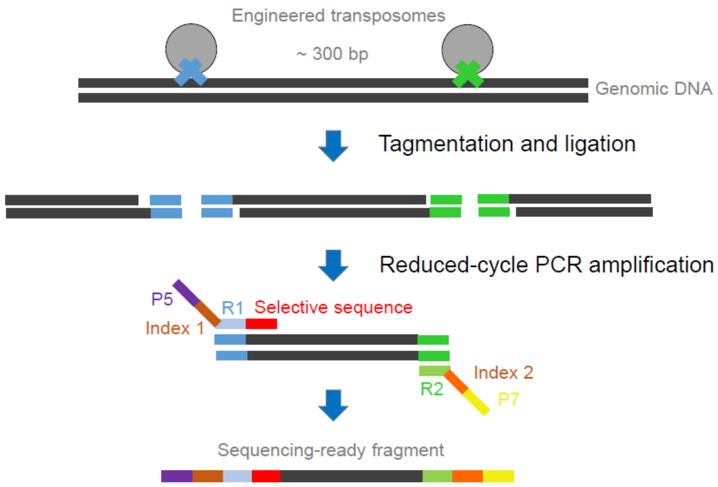

Supplement: S1 Fig — (PDF) [file pone.0177742.s001.pdf]
