# Supplementary material for: Using NextRAD sequencing to infer movement of herbivores among host plants
Source: PLoS One. 2017 May 15;12(5):e0177742. doi: 10.1371/journal.pone.0177742 (PMC5432177; doi:10.1371/journal.pone.0177742)

S2 Fig. Cross-validation (CV) error and standard error of ADMIXTURE runs of *K* ranging from 1 to 20. The lowest CV error was *K* = 9.

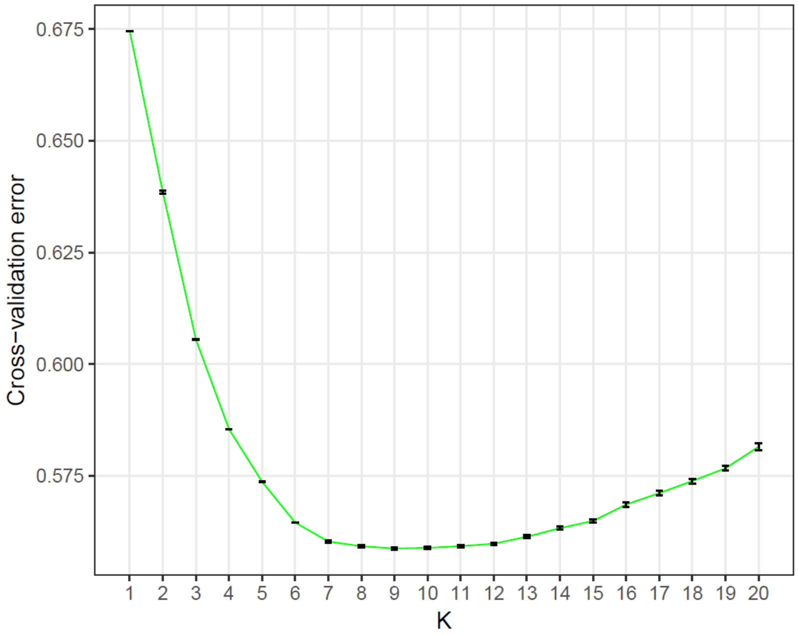

Supplement: S2 Fig — (PDF) [file pone.0177742.s002.pdf]
