# Supplementary material for: Using NextRAD sequencing to infer movement of herbivores among host plants
Source: PLoS One. 2017 May 15;12(5):e0177742. doi: 10.1371/journal.pone.0177742 (PMC5432177; doi:10.1371/journal.pone.0177742)

S3 Fig. Estimated ancestry of potato psyllids collected from bittersweet nightshade patches and potatoes. Potato psyllids were collected from bittersweet nightshade patches in (A) 2012, (B) 2013, and from potato fields in (C) 2013. Two *K*=2 plots were the next most represented grouping patterns over all 50 runs. Plots of *K*=8 and *K*=9 here were the most represented grouping patterns of *K* =8 and *K*=9 runs.

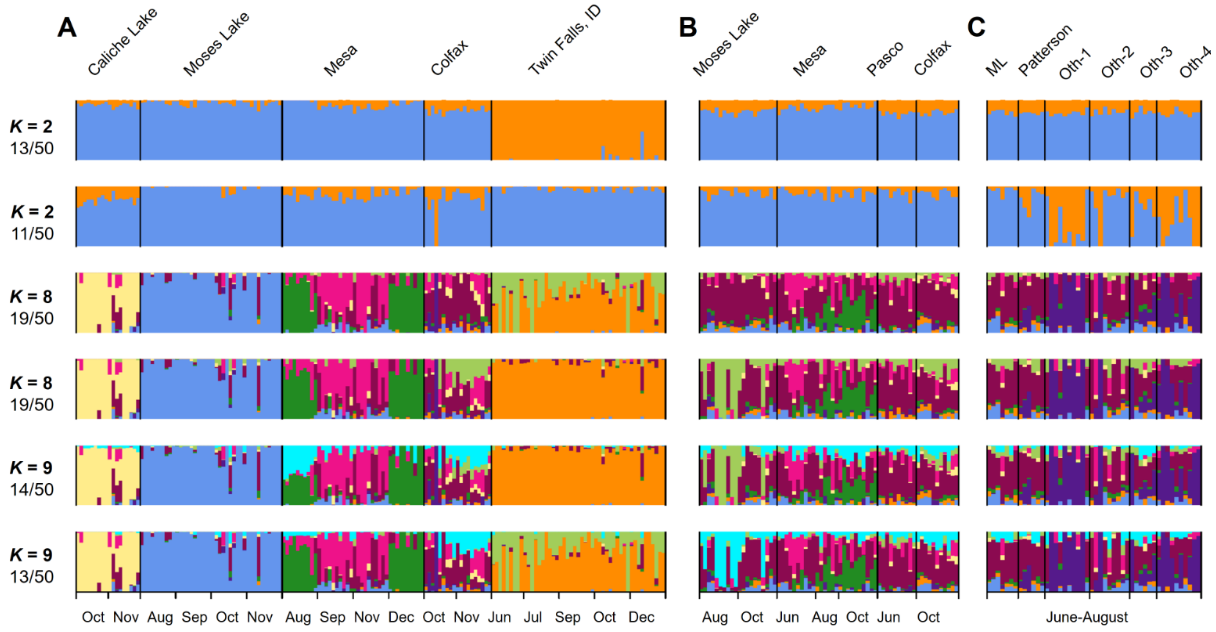

Supplement: S3 Fig — (PDF) [file pone.0177742.s003.pdf]
