# Supplementary material for: Using NextRAD sequencing to infer movement of herbivores among host plants
Source: PLoS One. 2017 May 15;12(5):e0177742. doi: 10.1371/journal.pone.0177742 (PMC5432177; doi:10.1371/journal.pone.0177742)

S4 Fig. Inbreeding coefficient, F_{IS} of psyllid populations collected from bittersweet nightshade patches (Nightshade) or from potato fields (Potato).

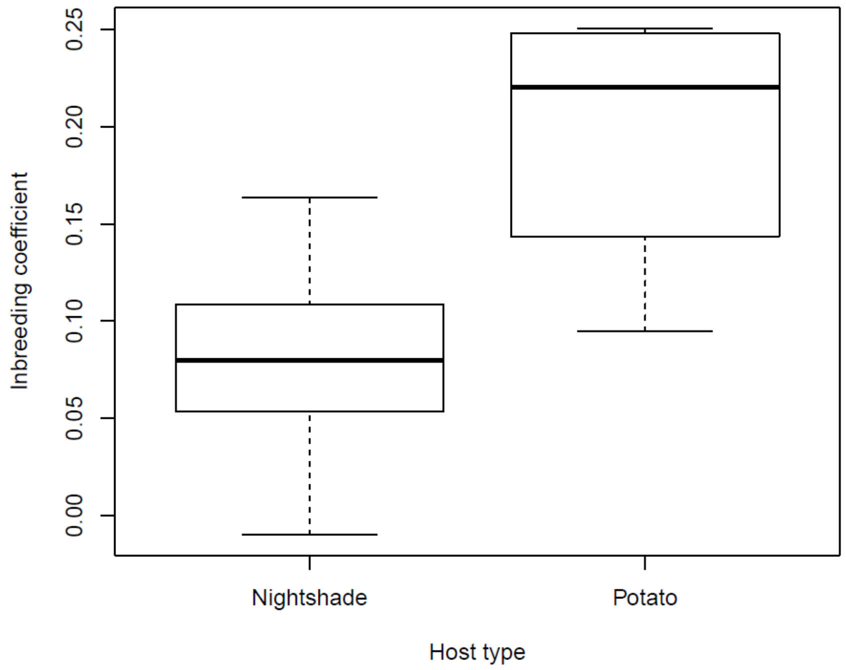

Supplement: S4 Fig — (PDF) [file pone.0177742.s004.pdf]
