# Supplementary material for: Using NextRAD sequencing to infer movement of herbivores among host plants
Source: PLoS One. 2017 May 15;12(5):e0177742. doi: 10.1371/journal.pone.0177742 (PMC5432177; doi:10.1371/journal.pone.0177742)

S5 Fig. Pairwise F_{ST} of psyllid populations separated on potato (Othello only) and nightshades shown in a heatmap. Sample name abbreviations from nightshades: CL=Caliche Lake, ML=Moses Lake, ME=Mesa, CX=Colfax, TF= Twin Falls, PA=Pasco, detailed sampling information were shown in Table 1 and S1 Table.

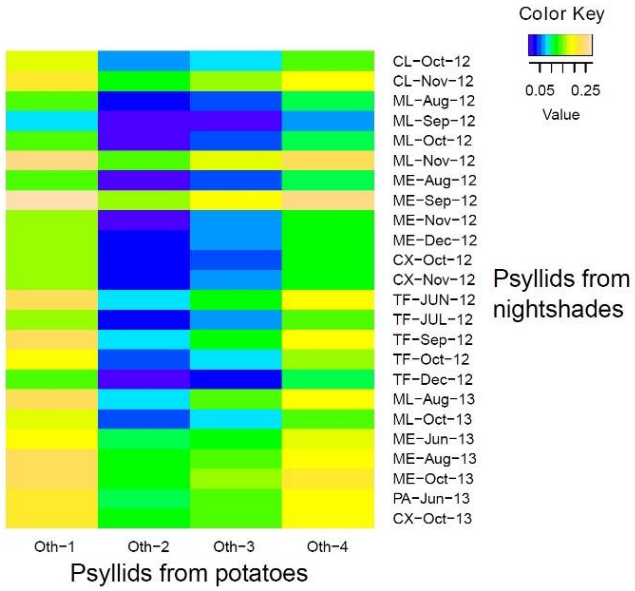

Supplement: S5 Fig — (PDF) [file pone.0177742.s005.pdf]
