# Supplementary material for: Using NextRAD sequencing to infer movement of herbivores among host plants
Source: PLoS One. 2017 May 15;12(5):e0177742. doi: 10.1371/journal.pone.0177742 (PMC5432177; doi:10.1371/journal.pone.0177742)
Supplement: S1 Table — (PDF) [file pone.0177742.s006.pdf]

S1 Table. Number of psyllids sampled from each sampling site at each date, for nightshade patches and potato fields in Washington and Idaho. Collections with sample size ≥ 4 were included in the *F*-statistics estimation (shaded in gray).

	2012							2013				
	Jun	Jul	Aug	Sep	Oct	Nov	Dec	Jun	Jul	Aug	Sep	Oct
**Nightshades**												
Caliche Lake (WA)					9	9						
Colfax (WA)					10*	10				1		10
Mesa (WA)			10	10		10	10	10		6		10
Moses Lake (WA)			10	10	10	10				10		10
Twin Falls (ID)	10*	10		10	10		10					
Pasco (WA)								10				
**Potatoes**												
ML-1									5			
ML-2									1			
ML-3										1		
Othello-1									1	10*		
Othello-2								1		9*		
Othello-3										6		
Othello-4										10		
Patterson-1										4		
Patterson-2									1			
Patterson-3										1		

* indicate a sample from that population was removed due to missing data
