# Supplementary material for: Using NextRAD sequencing to infer movement of herbivores among host plants
Source: PLoS One. 2017 May 15;12(5):e0177742. doi: 10.1371/journal.pone.0177742 (PMC5432177; doi:10.1371/journal.pone.0177742)
Supplement: S2 Table — (PDF) [file pone.0177742.s007.pdf]

S2 Table. Number of SNPs in each filtering step

From reads to SNPs	Number of SNPs
SNPs in initial genotyping	8443
[1]Removal of loci with > 5% missingness	2400
[2]Minor allele frequency (MAF) < 0.05	0
Removal of loci with Heterozygosity (Ho) > 0.5	923
Random select one SNP per contig	1978
HWE filter (p-value < 0.001)	0
SNPs used in population genetic analyses	1978

[1] We also removed four samples which had > 10 % of the missingness
[2] This filtering has been applied in the SNP calling process
