# Supplementary material for: Using NextRAD sequencing to infer movement of herbivores among host plants
Source: PLoS One. 2017 May 15;12(5):e0177742. doi: 10.1371/journal.pone.0177742 (PMC5432177; doi:10.1371/journal.pone.0177742)
Supplement: S3 Table — (PDF) [file pone.0177742.s008.pdf]

**S3 Table.** Pairwise population differentiation ($F_{ST}$) estimates of potato psyllid from nightshades at the Mesa site at different sampling dates: June (J), August (A), September (S), October (O), November (N) and December (D) of 2012 (12) and 2013 (13). * indicates *p*-value > 0.05, 500 bootstrapping was performed across loci.

| | A12 | S12 | N12 | D12 | J13 | A13 | O13 |
|---|---|---|---|---|---|---|---|
| A12 | - | 0.154 | 0.167 | 0.124 | 0.146 | 0.078 | 0.054 |
| S12 | | - | 0.002* | 0.185 | 0.019 | 0.034 | 0.049 |
| N12 | | | - | 0.185 | 0.015 | 0.028 | 0.053 |
| D12 | | | | - | 0.154 | 0.096 | 0.063 |
| J13 | | | | | - | 0.010 | 0.029 |
| A13 | | | | | | - | -0.011* |
| O13 | | | | | | | - |
