# Supplementary material for: Using NextRAD sequencing to infer movement of herbivores among host plants
Source: PLoS One. 2017 May 15;12(5):e0177742. doi: 10.1371/journal.pone.0177742 (PMC5432177; doi:10.1371/journal.pone.0177742)
Supplement: S4 Table — (PDF) [file pone.0177742.s009.pdf]

S4 Table. Pairwise population differentiation (F_{ST}) estimates of psyllids from the Moses Lake nightshade site at different sampling dates: August (A), September (S), October (O), November (N) of 2012 (12) and 2013 (13). * indicates *p*-value > 0.05, 500 bootstrapping was performed across loci.

	A12	S12	O12	N12	A13	O13
A12	-	0.018	0.005	0.001*	0.113	0.083
S12		-	0.017	0.006	0.116	0.086
O12			-	0.004*	0.088	0.053
N12				-	0.098	0.067
A13					-	0.022
O13						-
