# Supplementary material for: Using NextRAD sequencing to infer movement of herbivores among host plants
Source: PLoS One. 2017 May 15;12(5):e0177742. doi: 10.1371/journal.pone.0177742 (PMC5432177; doi:10.1371/journal.pone.0177742)
Supplement: S5 Table — (PDF) [file pone.0177742.s010.pdf]

S5 Table. Pairwise population differentiation (F_{ST}) estimates of potato psyllid from four potato fields at Othello in summer 2013. * indicates *p*-value > 0.05, 500 bootstrapping was performed across loci.

	Oth-1	Oth-2	Oth-3	Oth-4
Oth-1	-	0.096	0.037	-0.001*
Oth-2		-	0.013	0.066
Oth-3			-	0.014
Oth-4				-
