# Supplementary material for: Using NextRAD sequencing to infer movement of herbivores among host plants
Source: PLoS One. 2017 May 15;12(5):e0177742. doi: 10.1371/journal.pone.0177742 (PMC5432177; doi:10.1371/journal.pone.0177742)
Supplement: S6 Table — (PDF) [file pone.0177742.s011.pdf]

S6 Table. Median pairwise genetic distance (percent dissimilarity among individuals) and F_{ST} among groups of psyllids designated following ADMIXTURE $K = 3$ (Fig 3). While $K = 3$ in ADMIXTURE, there were four psyllids groups separated by hosts:

WA cluster 1 nightshade: psyllids in Washington from nightshades, shown as light blue bars in Fig 3 Panel A and B.

WA cluster 1 potato: psyllids in Washington from potatoes, shown as light blue bars in Fig 3 panel C.

WA cluster 3: psyllids collected from Othello (potato host) and one from Colfax (nightshade host), shown as purple bars in Fig 3 and labeled with red *.

Idaho: psyllids collected from Idaho shown as orange bars in Fig 3.

Groups	Median percent dissimilarity	F_{ST}
WA cluster 1: potato vs. nightshade	29	0.01
WA: potato cluster 1 vs. cluster 3	36	0.18
WA: nightshade cluster 1 vs. cluster 3	38	0.20
WA cluster 1 potato vs. Idaho	31	0.12
WA cluster 1 nightshade vs. Idaho	32	0.13
WA cluster 3 vs. Idaho	38	0.28
